# Microbiota-host metabolism reprogramming in colorectal cancer: from pathogenesis to precision therapies

**DOI:** 10.3389/fonc.2025.1713614

**Published:** 2025-12-03

**Authors:** Chengxu Guo, Caixia Wang

**Affiliations:** 1 Featured Laboratory for Biosynthesis and Target Discovery of Active Components of Traditional Chinese Medicine, School of Traditional Chinese Medicine, Binzhou Medical University, Yantai, China; 2 Collaborative Innovation Platform for Modernization and Industrialization of Regional Characteristic Traditional Chinese Medicine, School of Traditional Chinese Medicine, Binzhou Medical University, Yantai, China; 3 School of Clinical Medicine, Jining Medical University, Jining, China

**Keywords:** colorectal cancer, metabolic reprogramming, microbiota-derived metabolites, immunometabolism, precision therapy

## Abstract

Colorectal cancer (CRC) is a highly aggressive malignancy characterized by complex metabolic reprogramming, a hallmark that provides both biosynthetic precursors and signaling molecules to support tumor growth, invasion and therapeutic resistance. A key mechanism underlying this metabolic rewiring is the dynamic interplay between the host and gut microbiota. Gut microbiota derived metabolites, including short-chain fatty acids, secondary bile acids, polyamines and tryptophan derivatives, extensively reshape the CRC metabolic network and modulate the immune microenvironment, thereby influencing tumor progression and therapy response. This review systematically outlines the core features and molecular mechanisms of metabolic reprogramming in CRC, highlights the role of microbiota–host co-metabolism in regulating energy acquisition and immune-metabolic crosstalk, and discusses emerging therapeutic strategies that integrate metabolic targeting and microbiota modulation for precision intervention in CRC.

## 1 Introduction

CRC is one of the most common and lethal malignancies of the digestive system. Recent epidemiological data indicate that in 2022 there were approximately 1,930,000 new CRC cases worldwide, accounting for 9.6% of all cancers, and about 904,000 deaths, representing 9.3% of global cancer-related mortality. CRC ranks as the third most common cancer and the second leading cause of cancer-related death worldwide, surpassed only by lung cancer (1). Its pathogenesis is multifactorial, involving genetic mutations (2), chronic inflammation, tumor microenvironment (TME) remodeling (3) and dysbiosis of the gut microbiota (4). Metabolic reprogramming has been recognized as a new hallmark of cancer. Given the unique intestinal microbial ecosystem, CRC exhibits distinct modes of metabolic regulation (5). Traditionally, cancer metabolism research focused on the “Warburg effect,” wherein tumor cells preferentially utilize aerobic glycolysis even under oxygen-sufficient conditions. However, accumulating evidence demonstrates that CRC metabolic plasticity extends far beyond this single paradigm, encompassing multilayered remodeling of glucose, lipid, amino acid, nucleotide, and one-carbon metabolism. Such metabolic flexibility not only sustains cancer cell survival under hostile conditions but also fuels invasion, metastasis, and therapy resistance.

Gut microbes contribute to CRC pathogenesis through their metabolites or by secreting genotoxins (6). These microbiota-host co-metabolic pathways affect not only cancer cells but also the immune microenvironment, for instance, by modulating effector T cell activity and promoting immunosuppressive cell infiltration (7–9). Thus, understanding CRC from the dual perspective of tumor metabolism and microbial metabolism is essential to unravel its heterogeneity and provides a rationale for the development of precision therapeutic strategies, particularly those combining metabolic and immunomodulatory interventions (10). This review integrates recent advances to systematically examine the hallmarks of CRC metabolic reprogramming, the molecular mechanisms driving it, and the specific contributions of microbiota–host co-metabolism to tumorigenesis, therapy resistance, and immune evasion, while exploring the translational potential of these insights for future clinical applications.

## 2 Core features of metabolic reprogramming in CRC

Metabolic reprogramming has emerged as a fundamental hallmark of CRC, reflecting the ability of tumor cells to flexibly adapt their bioenergetic and biosynthetic demands to support malignant growth. Rather than functioning as isolated events, alterations in glucose, lipid, and amino acid metabolism constitute interdependent and highly dynamic networks that enable cancer cells to sustain proliferative signaling, resist cell death, and modulate the tumor microenvironment. These metabolic adaptations not only provide essential substrates for biomass accumulation but also participate in oncogenic signaling cascades and immune evasion, thereby linking metabolic plasticity to tumor progression and therapeutic resistance.

### 2.1 Reprogramming of glucose metabolism

Glucose metabolism is a central process for cellular energy acquisition, primarily occurring through three interconnected pathways: glycolysis, oxidative phosphorylation (OXPHOS), and the pentose phosphate pathway (PPP). CRC cells often exhibit the Warburg effect, relying predominantly on glycolysis to generate energy and metabolic intermediates even under aerobic conditions (11). Glycolysis occurs in the cytoplasm, where glucose enters the cell via transporters such as GLUT1, GLUT3, and GLUT4 (12). Hexokinase 2 (HK2) phosphorylates glucose to glucose-6-phosphate (G6P), preventing its efflux. G6P is then isomerized into fructose-6-phosphate (F6P) by phosphoglucose isomerase, followed by phosphorylation through phosphofructokinase-1 (PFK1) to form fructose-1,6-bisphosphate (F1,6BP). F1,6BP is cleaved by aldolase into glyceraldehyde-3-phosphate (G3P) and dihydroxyacetone phosphate (DHAP). Through subsequent enzymatic reactions, G3P is metabolized to generate NADH and ATP (13). Under hypoxia or metabolic preference of cancer cells, lactate dehydrogenase A (LDHA) converts pyruvate to lactate, which not only sustains redox balance but also preserves glucose availability for T cells, thereby enhancing antitumor immunity (14). A fraction of pyruvate enters the mitochondria, where it is converted by pyruvate dehydrogenase (PDH) into acetyl-CoA to fuel the TCA cycle. Within the TCA cycle, acetyl-CoA condenses with oxaloacetate to form citrate, which undergoes sequential enzymatic reactions to produce NADH, FADH^2^, and GTP (15, 16). NADH and FADH^2^ donate electrons to mitochondrial complexes I–IV, driving proton translocation across the inner mitochondrial membrane. The resulting electrochemical gradient powers ATP synthase (complex V) to generate large amounts of ATP (13). The PPP plays an indispensable role in antioxidant defense and biosynthesis in CRC. Unlike glycolysis and OXPHOS, PPP does not primarily produce ATP; instead, it generates NADPH and ribose-5-phosphate, which are essential for nucleotide synthesis, lipid biosynthesis, and reactive oxygen species (ROS) homeostasis (17, 18). This reprogramming is often accompanied by elevated expression of key glycolytic enzymes such as HK2, PKM2, and LDHA, which facilitate efficient glucose-to-lactate conversion. Simultaneously, PPP intermediates provide NADPH and nucleotides, supporting lipid synthesis, DNA repair, and redox balance (19). This coordinated reprogramming of glucose metabolism not only ensures a continuous energy supply and biosynthetic precursors for rapidly proliferating CRC cells but also establishes a metabolic niche that supports tumor progression, modulates the immune microenvironment, and contributes to therapeutic resistance.

### 2.2 Reprogramming of lipid metabolism

Lipid metabolism undergoes profound alterations in CRC. Both normal colonic epithelial cells and cancer cells are capable of generating ATP via fatty acid β-oxidation (FAO). However, CRC cells frequently exhibit upregulated lipogenic enzymes, including fatty acid synthase (FASN), acetyl-CoA carboxylase (ACC), and stearoyl-CoA desaturase 1 (SCD1), along with enhanced lipid uptake mediated by transporters such as CD36 and fatty acid binding proteins (FABPs). In glucose-limiting conditions, tumor cells rely heavily on FAO for ATP and NADPH production to sustain survival, proliferation, and invasion (18). Moreover, elevated levels of saturated fatty acids (SFAs) activate the TLR4/NF-κB signaling pathway, stimulating the secretion of pro-inflammatory cytokines such as TNF-α and IL-6. This promotes the establishment of a chronic inflammatory microenvironment, thereby driving CRC progression (20).

### 2.3 Reprogramming of amino acid metabolism

Glutamine metabolism is one of the most critical components of metabolic reprogramming in CRC, simultaneously fueling energy production, biosynthesis, and signal transduction. Glutamine is converted to glutamate by glutaminase (GLS), which is then metabolized by glutamate dehydrogenase (GLUD) to generate α-ketoglutarate (α-KG). α-KG enters the TCA cycle to replenish intermediates (anaplerosis), sustaining oxidative metabolism and energy generation (21). Beyond energy supply, α-KG serves as a carbon backbone for lipid, nucleotide, and non-essential amino acid synthesis. Meanwhile, glutamine-derived nitrogen contributes to purine and pyrimidine biosynthesis as well as transamination reactions (22, 23). Glutamine also fuels glutathione (GSH) synthesis, which is indispensable for detoxifying ROS and maintaining redox homeostasis. Under oxidative stress or chemotherapy-induced pressure, CRC cells often upregulate glutamine uptake to reinforce antioxidant capacity, thereby enhancing survival (24). Additionally, glutamine activates mTORC1 signaling to drive cell growth and protein synthesis. Myc upregulates glutamine transporters (e.g., SLC1A5/ASCT2) and GLS, further enhancing glutamine uptake and metabolism. CRC harboring KRAS mutations exhibit heightened glutamine dependency, particularly under glucose-limiting conditions (25). Competition for glutamine within the TME profoundly influences immune responses. Excessive glutamine uptake by cancer cells impairs the proliferation and effector functions of T cells, while glutamine depletion enhances the activity of immunosuppressive populations such as regulatory Tregs and myeloid-derived suppressor cells (MDSCs), ultimately promoting immune evasion (26). Given this reliance, GLS inhibitors are being investigated as potential therapeutic strategies to block glutamine utilization, disrupt energy supply, impair redox defense, and suppress tumor growth (24).

## 3 Key molecular mechanisms driving metabolic reprogramming

In CRC, oncogenic signaling pathways regulate the expression of metabolic enzymes through multiple molecular mechanisms, thereby reshaping the cellular metabolic landscape.

### 3.1 Wnt/β-catenin signaling and glycolysis

The Wnt/β-catenin pathway is one of the most central oncogenic drivers in CRC, often resulting from APC mutations that lead to abnormal accumulation of β-catenin. Recent studies have revealed that nuclear β-catenin not only regulates proliferation-associated genes but also directly enhances the expression of glycolytic enzymes such as PKM2 and GLUT1/SLC2A1. Spatial transcriptomics and clinical sample analyses have confirmed that the WNT7B/β-catenin axis activates the GLUT1 promoter, promoting glucose uptake and lactate accumulation, thereby establishing the Warburg phenotype. Functionally, this metabolic reprogramming increase proliferation and migration capacity and is closely associated with metastasis and poor prognosis. Thus, Wnt-driven glycolytic reprogramming is considered an early metabolic hallmark of CRC and a potential combination therapy target (27, 28).

### 3.2 PI3K-AKT-mTOR signaling and amplification of glycolysis

Mutations in PIK3CA and PTEN are common in CRC, resulting in constitutive activation of the PI3K-AKT-mTOR pathway. Over the past five years, evidence has shown that this pathway stabilizes HIF-1α and enhances c-Myc activity, thereby driving the expression of GLUT1, HK2, PFKFB3, and LDHA, which markedly amplifies glycolytic flux (29). In addition, mTORC1 activation stimulates ribosome biogenesis and purine synthesis, supplying precursors for rapid proliferation. Clinically, PI3K-mTOR inhibitors combined with glycolysis inhibitors exhibit stronger efficacy against therapy-resistant CRC models. This highlights that PI3K-AKT-mTOR-glycolysis coupling is not only an adaptive mechanism but also a therapeutic target (18, 30).

### 3.3 HIF-1α mediated hypoxic adaptation

Due to rapid tumor growth and uneven vascularization, CRC cells frequently experience hypoxia. Under such conditions, HIF-1α accumulate and translocate into the nucleus, directly upregulating GLUT1, HK2, PFKFB3, LDHA, and MCT4, thereby promoting high-flux glycolysis and lactate export (31). Recent studies show that excessive MCT4 expression not only clears lactate but also acidifies the tumor microenvironment, facilitating immune evasion, particularly by suppressing T-cell effector function. Clinical evidence demonstrates that high expression of HIF-1α and its metabolic targets correlates with metastasis and decreased survival. Targeting HIF-1α or MCT4 is thus considered a promising approach to enhance the efficacy of immune checkpoint inhibitors (32).

### 3.4 c-Myc and transcriptional metabolic remodeling

c-Myc is a classical oncogene in CRC, downstream of Wnt and EGFR signaling. Recent findings indicate a positive feedback loop between PKM2 and c-Myc: nuclear-localized PKM2 enhances Myc stability, while Myc upregulates PKM2 and other metabolic genes such as PDK and PRPS2, redirecting pyruvate toward lactate rather than mitochondrial oxidation and enhancing nucleotide biosynthesis. This mechanism is particularly prominent in metastatic CRC. Clinical data suggest that co-overexpression of c-Myc and PKM2 predicts chemotherapy resistance and poor prognosis. Breaking the c-Myc-metabolism positive feedback loop is therefore an important strategy for CRC combination therapies (33, 34).

### 3.5 Hippo/YAP-TAZ and glucose/lipid metabolism

Inactivation of the Hippo pathway results in aberrant activation of YAP/TAZ in CRC. Evidence shows that YAP1 directly binds the GLUT1 promoter and upregulates GLUT3, thereby increasing glucose uptake. Meanwhile, YAP/TAZ interact with SREBP to promote fatty acid and cholesterol biosynthesis (35). Functionally, this metabolic reprogramming enables tumor cells to sustain membrane synthesis and signal transduction under stress and is strongly linked to 5-FU resistance. Clinical analyses indicate that high YAP expression significantly correlates with reduced survival in CRC patients (36, 37).

### 3.6 SREBP1/2-driven lipid biosynthesis

SREBPs are master transcription factors of lipid metabolism. In CRC, SREBP1 upregulates FASN, ACACA, and SCD1 to drive fatty acid synthesis, whereas SREBP2 activates HMGCR and HMGCS1 to promote cholesterol biosynthesis. Recent mass spectrometry-based metabolomics evidence demonstrates markedly enhanced lipid metabolism in CRC tissues, particularly in the context of KRAS mutations (38). High-fat diet or obesity further amplifies this axis, underscoring its role in gene-environment interactions. Pharmacological inhibition of FASN or HMGCR reduces CRC growth and increases chemosensitivity (28).

### 3.7 KRAS-driven glutamine dependence

CRC harboring KRAS mutations exhibit “metabolic addiction” to glutamine. Mechanistically, KRAS activates transcription and stabilizes GLS1/2 and GOT2, facilitating glutamine catabolism to α-ketoglutarate, which replenishes the TCA cycle and supports glutathione synthesis to counteract oxidative stress. Animal and organoid models show that the GLS inhibitor CB-839 exerts enhanced antitumor effects in KRAS-mutant CRC, particularly when combined with energy metabolism inhibitors such as metformin (39, 40).

### 3.8 ATF4 and the serine synthesis pathway

Under amino acid deprivation or cellular stress, ATF4 translocate into the nucleus and directly activates PHGDH, PSAT1, and PSPH, driving the serine synthesis pathway (SSP). Serine not only contributes to protein biosynthesis but also feeds into one-carbon metabolism, supporting DNA synthesis and methylation. Clinical studies in CRC have shown that patients with high ATF4-SSP expression exhibit increased resistance to 5-FU. Moreover, the transcription factor FOXC1 enhances SSP and promotes DNA repair. Targeting PHGDH or restricting dietary serine/glycine has been proposed as a metabolic intervention strategy for CRC (41).

CRC metabolic reprogramming is driven by multiple interconnected mechanisms. These processes are shaped not only by organ- and spatial-level heterogeneity within CRC but also by tight interactions with gut microbiota-host co-metabolism, together constructing a metabolic-immune evasion network. This has profound implications for precision therapy and prognostic prediction in immunotherapy.

## 4 Spatial, tissue, and organ heterogeneity of metabolic reprogramming

Metabolic reprogramming in CRC exhibits pronounced spatial and tissue heterogeneity. Spatial transcriptomics has revealed that the tumor core displays higher activation of nucleotide metabolism, whereas the peripheral regions are enriched in pathways related to cell–cell interaction and migration (42). Single-cell and spatial multi-omics further demonstrate that epithelial cells, stromal cells, and immune cells within the same lesion form multiple metabolic subprograms, driving clonal evolution and heterogeneity in therapeutic responses (43, 44).

Cross-cell metabolic coupling represents a key mechanism underlying spatial heterogeneity. Cancer-associated fibroblasts (CAFs) and tumor cells engage in a “lactate shuttle,” redistributing carbon flux to promote invasion, angiogenesis, and immune suppression. This phenomenon has been validated in both *in vitro* and *in vivo* CRC models as well as in multi-cohort analyses, and has been extended to a broader framework where lactate signaling or histone lactylation influences immunity and drug resistance (45–47).

At the tissue level, right-sided and left-sided CRC differ not only in molecular subtypes and immune landscapes but also in metabolic profiles, which display a continuous gradient along the gastrointestinal axis rather than a simple “right-left” dichotomy. This gradient provides a basis for prognostic assessment and stratified treatment strategies (48, 49).

The metabolic phenotype of CRC is characterized not only by intratumoral spatial heterogeneity but also by organ-specific adaptations between primary tumors and metastatic lesions. In general, metastases do not simply “replicate” the primary tumor; instead, they undergo selective adaptation and remodeling according to the metabolic milieu of the host organ, including substrate availability, redox state, local immunity, stromal composition, and cavity fluid dynamics. For instance, colorectal liver metastases (CRLM) frequently exhibit enhanced cholesterol biosynthesis and sterol regulatory element-binding protein (SREBP)-dependent pathways, leveraging the lipid-rich hepatic microenvironment to support rapid proliferation and lipid signaling. Moreover, the immune-metabolic interactions in the liver (e.g., with Kupffer cells and hepatic stellate cells) create an immunosuppressive and drug-resistant metabolic landscape (50). By contrast, lung metastases often preferentially activate fatty acid metabolism and antioxidant responses-such as through ATP citrate lyase (ACLY) to counteract the relatively high oxidative stress of pulmonary tissue while sustaining bioenergetic demands (51, 52). Peritoneal metastases face challenges of low adhesion support, stromal metabolic constraints, and niche limitations. To achieve colonization and chemotherapy tolerance, tumor cells rely on lipid storage, amino acid alternative metabolism, and metabolic coupling with peritoneal mesothelial and immune cells. These features partly explain the poor prognosis and reduced treatment sensitivity observed in peritoneal metastases (53). Rare bone or bone marrow metastases depend on bone marrow adipocytes and bone remodeling-related metabolic signals (eg. lipid metabolic regulation, RANKL/CSF-1 pathways) to provide an energy-supportive microenvironment for cancer cell survival (54).

In addition, tumor-derived exosomes and amino acid metabolism pathways, such as glutamine and aspartate metabolism, play critical roles in organ-specific metabolic reprogramming (55). Therefore, identifying and targeting “site-specific” metabolic vulnerabilities rather than relying on generalized metabolic inhibition holds greater promise for achieving therapeutic benefit across distinct metastatic niches.

## 5 Microbiota-host co-metabolism in CRC

The gut microbiota and its metabolites can modulate immune cell activity via direct and indirect mechanisms. Cameron et al. demonstrated that *Bacteroides fragilis* induces proliferation of natural killer T (NKT) cells in mice and promotes their differentiation into IL-10 producing immunoregulatory cells, thereby enhancing immune activity (56). Moreover, bacterial-driven immune stimulation supports the formation of memory T cells, which are essential for maintaining long-term antitumor immunity and reducing tumor recurrence risk.

Within the TME, oxygen availability, lactate accumulation, extracellular pH, and nutrient gradients synergistically shape metabolic heterogeneity. Hypoxia, acidosis, and nutrient competition drive tumor cell metabolic reprogramming. Hypoxia-inducible factor-1α (HIF-1α) upregulates glycolytic gene expression under low-oxygen conditions, while lactate buildup lowers extracellular pH, suppressing effector T-cell and dendritic cell (DC) function and facilitating the recruitment of immunosuppressive Tregs and M2 macrophages, thereby fostering an immune-privileged environment (26).

**Figure 1:**
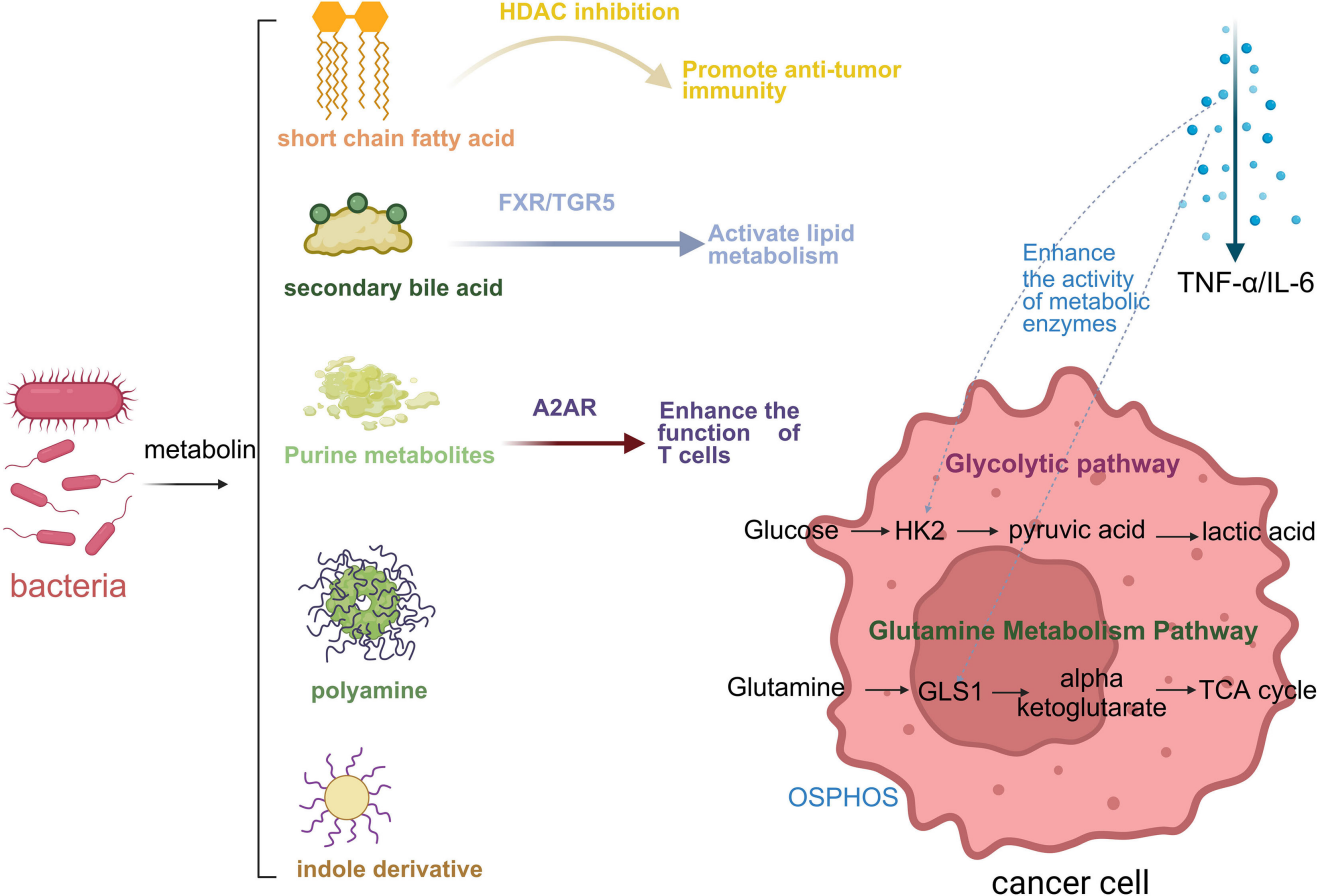
The microbiota-host co-metabolic pathway deeply shapes the metabolic network of CRC. Schematic illustration of microbiota-derived metabolites regulating cancer cell metabolism and immune responses. Short-chain fatty acids, secondary bile acids, purine metabolites, polyamines, and indole derivatives modulate host signaling pathways, influencing glucose and glutamine metabolism in cancer cells. These interactions affect glycolysis, the TCA cycle, and inflammatory cytokine release (TNF-α/IL-6), thereby shaping tumor progression and anti-tumor immunity.

The gut microbiota profoundly influences colorectal epithelial cell proliferation, DNA integrity, inflammation and immune responses, epigenetic states, and nutrient availability within the microenvironment through its metabolites-short-chain fatty acids (SCFAs), secondary bile acids, tryptophan- and indole-derived compounds, trimethylamine N-oxide (TMAO), polyamines, and microbial toxins such as colibactin as well as through metabolic interactions including cross-feeding and substrate competition (57, 58) (**Figure 1**).

### 5.1 Microbiota-derived metabolites

Microbiota-host co-metabolism plays a pivotal role in the initiation and progression of CRC, as revealed by recent metabolomics and mechanistic studies. SCFAs, especially butyrate, are produced by bacterial fermentation of dietary fiber. In colonic epithelial cells, butyrate serves both as an energy substrate and as an inhibitor of histone deacetylases (HDACs), thereby altering gene expression, inducing differentiation, suppressing aberrant proliferation, and strengthening epithelial barrier integrity and local immune responses (e.g., promoting regulatory T cells and enhancing antitumor immunity) (59). However, in “Warburg-like” tumor cells where butyrate utilization is restricted, its accumulation exerts stronger HDAC inhibition and consequently displays pronounced antitumor activity (58, 60). In contrast, secondary bile acids (e.g., deoxycholic acid, deoxycholic acid (DCA) primarily exert carcinogenic effects. Primary bile acids undergo microbial 7α-/7β-dehydroxylation in the gut to form DCA, which induces oxidative stress, DNA damage, and chronic inflammation, while activating proliferative signals such as EGFR/STAT3 to promote tumorigenesis. Metabolomic evidence shows elevated levels of certain secondary bile acids in the feces and circulation of CRC patients, significantly associated with cancer risk and progression (61, 62).

Beyond fatty acids and bile acids, tryptophan-derived metabolites represent another key interface of microbiota-host crosstalk. Microbiota convert tryptophan into various indole derivatives (e.g., indole-3-propionic acid, indole-3-acetic acid, and other aromatic compounds), which act as ligands for the aryl hydrocarbon receptor (AhR), regulating immune homeostasis, promoting epithelial repair, and maintaining barrier function (63). By contrast, the host IDO/TDO-mediated kynurenine pathway contributes to immunosuppression by driving regulatory T-cell differentiation and effector T-cell exhaustion, thereby fostering tumor immune evasion and presenting a “double-edged sword” effect (64). In parallel, microbiota-derived toxins exert direct oncogenic pressure, with colibactin produced by pks+ *Escherichia coli* as a representative example. Upon contact with colonic epithelium, colibactin induces DNA adducts and double-strand breaks. Chronic exposure results in characteristic mutational signatures (e.g., SBS88/ID18). Large-scale genomic sequencing has recently identified colibactin-associated mutational fingerprints in numerous CRC specimens, underscoring that such microbial genotoxins are not incidental but actively contribute to carcinogenesis (59, 65).

Other emerging metabolic pathways are also gaining attention. For instance, microbiota-derived trimethylamine (TMA) is converted in the host liver to TMAO, which though its exact role in CRC remains under investigation is linked to systemic inflammation and risk of other cancers. Polyamines (e.g., putrescine, spermidine), co-produced by microbiota and host, participate in cell proliferation, metabolic regulation, and immune remodeling within the tumor microenvironment (66). In addition, microbiota-modified lipid metabolites may influence local inflammation and tumor susceptibility through fatty acid receptor mediated signaling pathways.

### 5.2 Key mechanistic layers of microbiota-host metabolic interaction

Microbiota–host metabolic crosstalk in CRC operates through multiple hierarchical and interconnected mechanisms. First, at the direct metabolic (substrate-product) level, gut microbes transform dietary components such as fiber, cholesterol, and amino acids into bioactive molecules. Once absorbed by colonic epithelial cells, these metabolites directly influence host metabolic pathways, gene expression, and DNA stability (57). For example, butyrate, among SCFAs, inhibits HDAC activity, thereby modulating transcription to suppress proliferation and promote differentiation (67), whereas secondary bile acids such as DCA induce DNA damage and chronic inflammation, driving tumorigenesis (60, 68).

Second, at the receptor/signaling transduction level, many microbial metabolites activate host pathways via specific receptors, thereby shaping local and systemic homeostasis. SCFAs signal through G-protein-coupled receptors (GPR41/GPR43, also known as FFAR3/2, and GPR109A) to regulate immune responses and epithelial barrier function (69), indole-derived tryptophan metabolites modulate immune cell differentiation and mucosal defense via AhR, while secondary bile acids signal through FXR and TGR5 to regulate energy metabolism and inflammation (70, 71). Such receptor-mediated signaling is considered a core mechanism through which microbial metabolites exert long-term, systemic effects on the host.

Third, metabolism-epigenetics coupling provides a deeper mechanistic layer for microbial metabolites to reprogram host cells. Multiple metabolites including lactate, SCFAs, and S-adenosylmethionine derivatives modulate histone acetylation, methylation, and DNA methylation patterns, durably reshaping transcriptional programs in epithelial and immune cells (72). For example, the HDAC inhibitory effect of butyrate not only suppresses tumor cell proliferation but also establishes persistent “metabolic memory,” driving long-term anticancer gene expression profiles and highlighting its critical role in chronic tumor suppression (73).

Finally, spatial heterogeneity of microbial communities and metabolic fluxes represents another key dimension of CRC metabolic reprogramming. Distinct microbial compositions and activities exist across the gut lumen, mucosal surface, and mucus layer, leading to unique metabolite concentration gradients at different spatial locations. Recent advances in spatial omics and metabolic imaging have revealed specific “metabolic niches” around tumors, where local concentrations of metabolites such as DCA or butyrate differ markedly from adjacent normal tissues (74). This spatial heterogeneity not only influences tumor cell metabolic states but also reshapes interactions between immune cells and stromal components, thereby driving or restraining tumor progression at the microenvironmental level.

### 5.3 Microbial role in nutrient acquisition

The gut microbiota directly participates in host nutrient acquisition and utilization through multiple metabolic pathways, thereby profoundly reshaping the metabolic landscape that underlies CRC initiation, progression, and therapeutic response. Specifically, microbes ferment indigestible dietary fibers into short-chain fatty acids (SCFAs, particularly butyrate), which serve as the preferred energy substrates for normal colonic epithelial cells (75), supporting epithelial homeostasis and repair. At the same time, SCFAs act as epigenetic regulators (HDAC inhibitors) that reprogram gene expression, promote differentiation, suppress aberrant proliferation, and modulate local immunity (including effects on regulatory and effector T cells). Thus, in early tumorigenesis, butyrate often exerts protective effects. However, in tumor cells that have undergone a Warburg-type metabolic shift, butyrate utilization is restricted, leading to its accumulation and a stronger HDAC-inhibitory effect that turns toward antitumor activity (76).

Meanwhile, gut microbes chemically modify bile acids via 7α/7β-dehydroxylation, converting liver-derived primary bile acids into secondary bile acids such as DCA. These metabolites induce oxidative stress, inflammation, and DNA damage, while activating proliferative signaling pathways (e.g., EGFR/STAT3), thereby increasing epithelial carcinogenic risk and altering host metabolism of lipids and fat-soluble nutrients. Population-based and metabolomics studies have repeatedly detected CRC-associated alterations in secondary bile acid profiles in patient feces and blood (77).

With respect to amino acid metabolism, gut microbes can both degrade dietary proteins to release absorbable nitrogen sources and synthesize or convert amino acids (e.g., tryptophan metabolism into indole derivatives) to produce small signaling molecules. Microbiota-derived indole ligands act on host receptors such as AhR to regulate epithelial repair, barrier function, and immune homeostasis; meanwhile, the host IDO/TDO–kynurenine axis can be modulated by microbial metabolic activity, contributing to immunosuppression and tumor immune evasion. Moreover, microbial-to-host amino acid/indole fluxes can provide anaplerotic input to the TCA cycle or regulate antioxidant capacity, thereby supporting tumor proliferation and drug resistance (78).

Additionally, gut microbes synthesize B-vitamins and other cofactors (e.g., vitamin B6, folate, B12), influencing one-carbon (1-C) metabolism and methyl donor balance, which in turn impacts nucleotide biosynthesis, DNA methylation, and epigenetic programming. This “microbiota → vitamin → 1-C metabolism” axis has been proposed to regulate tumor growth rates and modulate chemotherapy sensitivity (79).

### 5.4 Microbial metabolism and therapy

Microbial metabolism plays a dual and pivotal role in CRC therapeutic responses enhancing efficacy in some contexts while driving resistance or toxicity in others. First, microbiota-modified secondary bile acids can suppress local cytotoxic immune responses within tumors, reducing CD8^+^ T-cell activity and thereby diminishing the efficacy of immune checkpoint inhibitors (ICIs). These metabolites are enriched in the gut and tumor microenvironment of CRC patients and have been shown to promote tumor growth by impairing effector T-cell function (61).

Second, the impact of SCFAs (particularly butyrate) on therapy is context-dependent. In some models, butyrate from the gut lumen or tumor microenvironment enhances the cytotoxic effects of chemotherapeutics such as 5-FU. However, recent studies indicate that butyrate can also promote resistance to anti-PD-1 therapy or certain chemotherapies by upregulating CPT1A-mediated fatty acid oxidation (FAO) or activating PI3K/AKT signaling, suggesting that its spatial distribution and concentration determine the ultimate therapeutic outcome (80, 81).

Microbial enzymatic activity directly influences chemotherapy toxicity and drug metabolism. For example, in irinotecan treatment, microbial β-glucuronidase (GUS) reactivates the hepatically detoxified metabolite SN-38-glucuronide back to active SN-38, leading to severe intestinal toxicity. Small-molecule inhibitors of GUS or targeted microbiota modulation have shown significant efficacy in preclinical and early studies in reducing toxicity and improving treatment tolerance (82). Moreover, certain microbial metabolites or toxins (e.g., colibactin) induce characteristic mutational signatures (SBS88/ID18) that stably alter the tumor genome. Such effects not only contribute to carcinogenesis but may also influence tumor sensitivity to DNA-damaging therapies (e.g., radiotherapy, certain chemotherapeutics) or facilitate the emergence of resistance-associated mutations. Recent tumor genomics studies have detected colibactin-associated mutational fingerprints in clinical samples, underscoring their potential relevance for therapeutic decision-making (83).

## 6 Regulation of the immune microenvironment by microbial metabolites

Gut microbiota–derived metabolites play pivotal roles in shaping the immune microenvironment of CRC, involving multiple immune signaling pathways, metabolic regulatory networks, and intercellular interactions.

### 6.1 Immunosuppressive microbial metabolites in the TME

First, secondary bile acids such as DCA and lithocholic acid (LCA), generated from primary bile acids through bacterial 7α-dehydroxylation and related reactions, exert profound immunomodulatory effects. These metabolites activate the nuclear receptor farnesoid X receptor (FXR) and the membrane receptor TGR5, thereby upregulating the expression of the immunosuppressive cytokine IL-10 and promoting regulatory T cell (Treg) differentiation, ultimately reinforcing immune tolerance. In addition, DCA and LCA suppress dendritic cell (DC) maturation and antigen-presenting capacity, diminish natural killer (NK) cell cytotoxicity, and drive tumor-associated macrophages (TAMs) toward an immunosuppressive M2-like phenotype. Collectively, these changes create a TME conducive to immune evasion (70, 71).

Second, polyamines such as spermidine and putrescine are markedly elevated in the gut and serum of CRC patients, derived from dietary sources, host synthesis, and microbial metabolism (e.g., amino acid decarboxylation by *Escherichia coli* and *Bacteroides*). Polyamines suppress T cell receptor (TCR) signaling and downregulate the adhesion molecule CD44, thereby reducing T cell–target cell interactions and cytotoxic efficiency. They also decrease secretion of proinflammatory cytokines IFN-γ and TNF-α, impairing T and B lymphocyte activation and proliferation. Beyond direct immunosuppression, polyamines modulate extracellular matrix remodeling via tumor-associated stromal cells, indirectly fostering an immunosuppressive TME (84).

**Figure 2:**
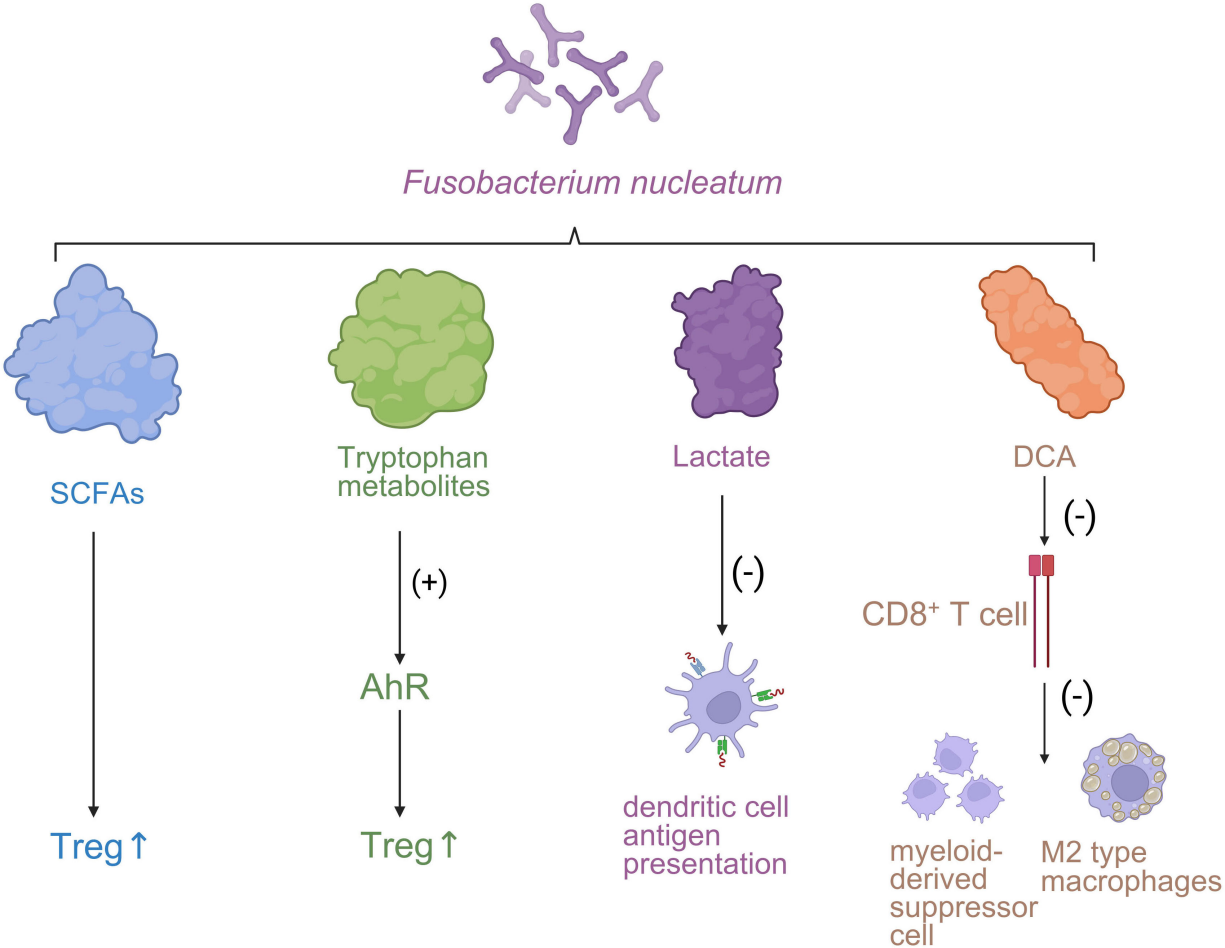
The influence of the immune microenvironment on the progression of CRC. *Fusobacterium nucleatum*-derived metabolites modulate immune responses in the tumor microenvironment. SCFAs and tryptophan metabolites enhance regulatory T cell (Treg) activity, while lactate suppresses dendritic cell antigen presentation. Deoxycholic acid (DCA) inhibits CD8^+^ T cell function and promotes immunosuppressive myeloid-derived suppressor cells (MDSCs) and M2-type macrophages.

Lactate, the predominant metabolite of aerobic glycolysis (Warburg effect) in tumor cells, accumulates to high levels in the hypoxic, glucose-rich TME. Lactate enters tumor-associated neutrophils (TANs) via the monocarboxylate transporter MCT1, activating the NF-κB/COX-2 signaling axis and inducing expression of the immune checkpoint molecule PD-L1, thereby directly suppressing CD8^+^ cytotoxic T lymphocyte (CTL) activity. Moreover, lactate impairs DC maturation and limits antigen presentation, further promoting immune evasion and metabolic immunosuppression (85, 86) (**Figure 2**).

### 6.2 The dual immunomodulatory roles of short-chain fatty acids

Short-chain fatty acids (SCFAs), particularly butyrate and propionate, are key products of dietary fiber fermentation by gut microbiota. SCFAs regulate immunity by inhibiting histone deacetylases (HDACs) and activating G protein-coupled receptors (GPR43, GPR109A, among others), promoting Treg differentiation and secretion of immunosuppressive cytokines IL-10 and TGF-β, while suppressing Th17 and DC differentiation and reducing proinflammatory cytokine production (e.g., TNF-α, IL-6). Notably, under specific immune contexts, butyrate not only exerts immunosuppressive effects but also enhances the antitumor activity of CD8^+^ T cells. Mechanistically, this involves HDAC inhibition mediated activation of ID2-dependent IL-12 signaling, leading to upregulation of IFN-γ, TNF-α, and CD25 expression, which in clinical models augments the efficacy of CAR-T cell therapy and chemotherapeutics such as oxaliplatin (87, 88). Additionally, SCFAs activate the mTOR/STAT3 pathway and NLRP3 inflammasome via GPCR signaling, stimulating IL-18 and antimicrobial peptide secretion, thereby strengthening intestinal barrier integrity and immune homeostasis.

In innate immune regulation, SCFAs exhibit bidirectional effects on macrophages and neutrophils: on one hand, they inhibit HDAC activity, reducing expression of proinflammatory mediators (IL-12, NO, TNF-α); on the other hand, their role in M2 macrophage polarization remains debated. Some studies report SCFAs suppress M2 polarization, thereby enhancing antimicrobial and antitumor functions, whereas others suggest that in tissue repair contexts SCFAs may sustain M2-like traits (89). By contrast, the immunosuppressive effect of lactate on neutrophils is well established: through MCT1-mediated signaling, lactate persistently induces PD-L1 expression, reinforcing CTL dysfunction in a vicious cycle (90).

In mucosal immunity, propionate acts directly on intestinal γδT cells, inhibiting their secretion of IL-17 and IL-22 through HDAC inhibition, thereby reducing Th17-type immune responses, alleviating excessive inflammation, and maintaining intestinal immune homeostasis (91).

**Figure 3:**
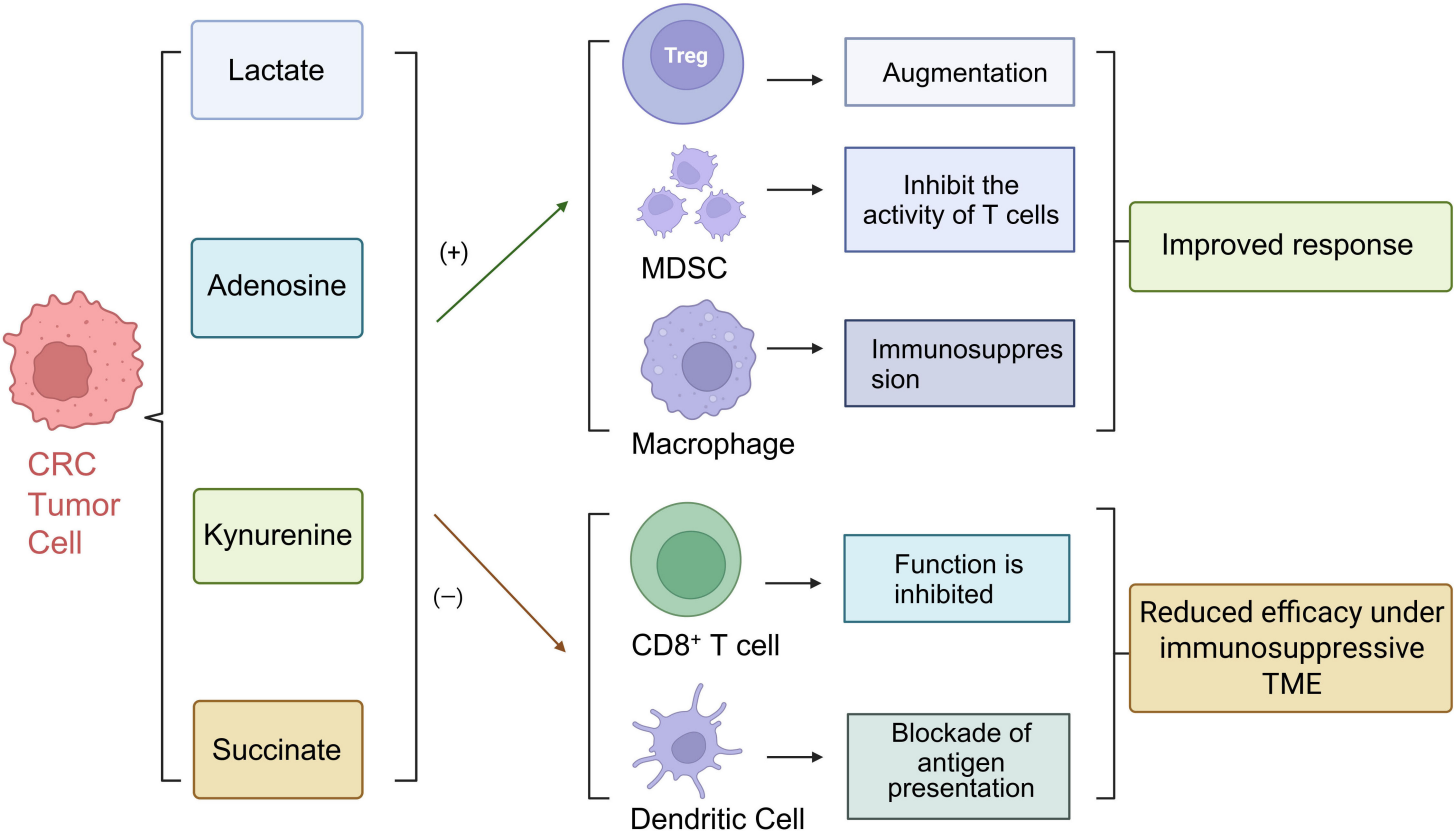
The influence of the immune microenvironment on the therapeutic response of CRC. Immunomodulatory effects of CRC tumor cell-derived metabolites. Lactate, adenosine, and kynurenine enhance regulatory T cells (Tregs), myeloid-derived suppressor cells (MDSCs), and macrophage-mediated immunosuppression, leading to improved tumor adaptation. In contrast, succinate and related intermediates inhibit CD8^+^ T cell function and block dendritic cell antigen presentation, resulting in reduced therapeutic efficacy under an immunosuppressive tumor microenvironment (TME).

In summary, microbial metabolites exert profound regulatory effects on both adaptive and innate immune cells through receptor-mediated signaling, epigenetic modifications, and metabolic reprogramming. This dual functionality can either suppress inflammation and preserve tissue homeostasis or facilitate immune escape and tumor progression within the TME. Consequently, precision targeting of specific microbial metabolites represents a promising strategy for CRC immunotherapy and microenvironmental remodeling (**Figure 3**).

## 7 Therapeutic strategies targeting metabolism and microbiota

**Figure 4:**
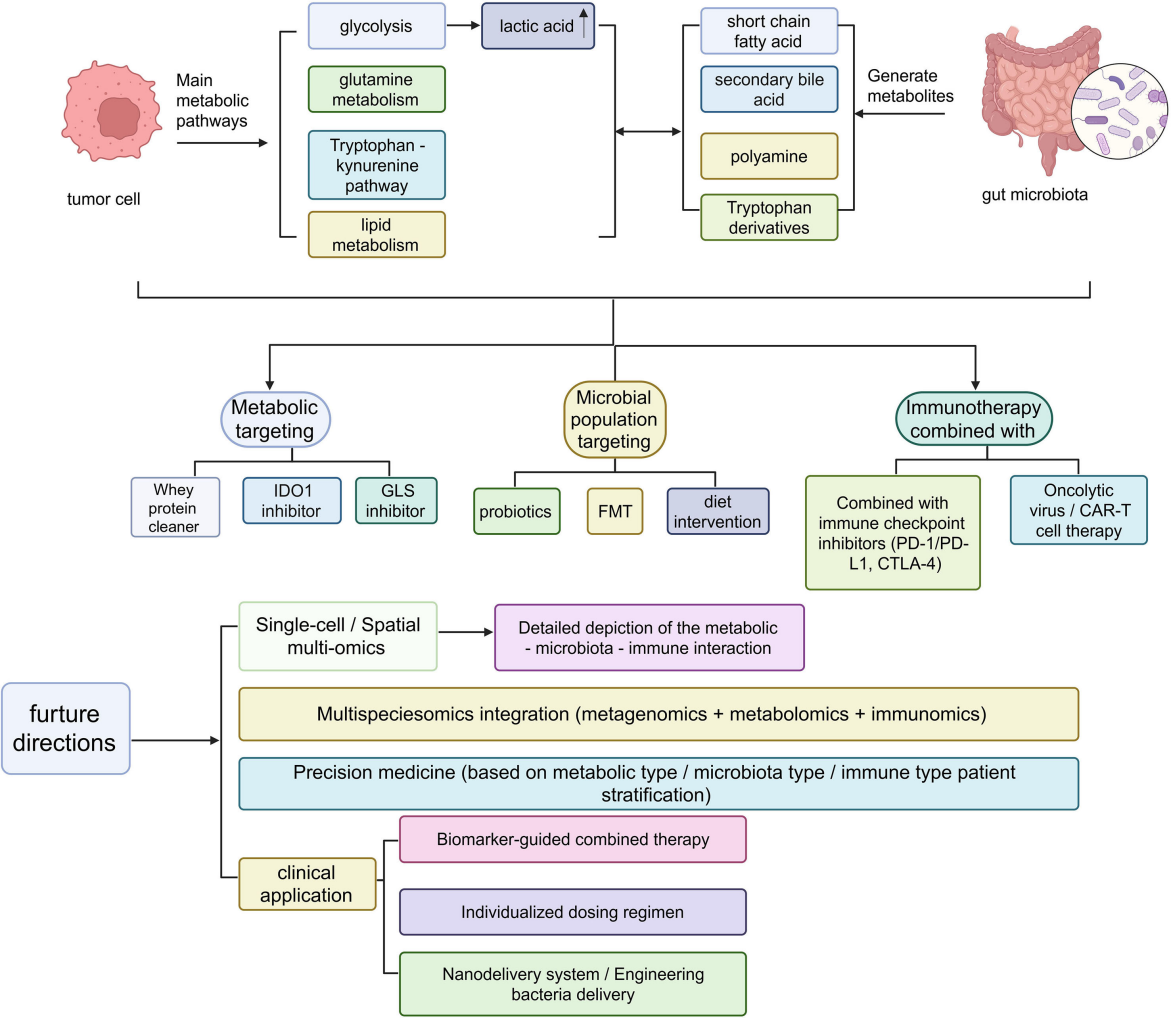
CRC adopts a combined intervention strategy based on metabolism and microbiota targeting, as well as the future research directions for transformation. Metabolic-microbiota-immune crosstalk in cancer therapy. Schematic overview of key microbial and host metabolic pathways (glycolysis, tryptophan-kynurenine, polyamine, bile acid, short-chain fatty acids) and their therapeutic targeting via diet, probiotics, FMT, oncolytic viruses, CAR-T, checkpoint inhibitors, and nanodelivery systems, highlighting multi-omics integration toward precision-immunotherapy stratification.

With the rapid advances in tumor metabolomics and gut microbial ecology, integrative therapeutic strategies that combine targeting of metabolic pathways with microbiota modulation are emerging as an important direction for precision medicine in CRC. This approach not only aids in early diagnosis and subtyping but also optimizes therapeutic target selection, helps overcome drug resistance, enhances responses to immunotherapy, and, to some extent, reduces the toxic side effects of conventional treatments (**Figure 4**).

### 7.1 Diagnosis and subtyping

In recent years, numerous studies have demonstrated that gut microbiota and their derived metabolites undergo stable and reproducible alterations during the initiation and progression of CRC, making them promising predictive tools for diagnosis and disease stratification. A large-scale meta-analysis integrating 18 cohorts and 3,741 samples revealed that fecal microbiota, at both the strain and functional pathway levels, can be utilized for CRC screening and progression prediction, and indicated that distinct functional groups and bacterial strains are associated with different disease stages (92). At the level of specific microbial signatures, *Fusobacterium nucleatum* has been widely reported to correlate with CRC detection rates, prognosis, and metastatic risk. It can enhance predictive model performance both as a single biomarker and in combination with clinical parameters (93, 94). Moreover, recent findings highlight significant remodeling of archaeal communities in CRC, along with interactive changes within bacterial networks, suggesting that integrating “bacteria + archaea” may further improve diagnostic and subtyping accuracy (95). Additionally, anatomical tumor location has been shown to influence microbial signatures, for example, differences in fecal microbiota between proximal and distal CRC may reduce model performance underscoring the need for location-specific stratification in clinical applications (96).

Beyond microbial features, metabolites as functional readouts also exhibit strong predictive value. Metabolomic analyses of fecal and plasma samples have demonstrated that amino acid metabolites, bile acid subtypes, tryptophan derivatives, and polyamines can robustly differentiate CRC patients from healthy controls, while also displaying directional changes along the adenoma-to-carcinoma continuum (97). Prospective cohort studies and systematic reviews further suggest that some metabolites may serve as risk assessment biomarkers prior to clinical onset, highlighting their potential in early detection. Importantly, integrating microbiome and metabolomic data allows the identification of functional metabolic pathways strongly linked to CRC progression and significantly enhances the sensitivity and specificity of diagnostic models (98). Several studies have proposed that metagenomic testing using fecal immunochemical test (FIT) sampling tubes is feasible, enabling seamless integration into existing screening systems and thus improving clinical applicability (99).

In addition, metabolic imaging and metabolomic technologies are providing novel tools for CRC subtyping and personalized treatment. Positron emission tomography (PET) can detect tumor glucose uptake and metabolic activity by tracking radiolabeled glucose analogs. Studies indicate that CRCs with high glucose metabolism often exhibit elevated standardized uptake values on PET scans, with concurrent upregulation of hexokinase 2 and lactate dehydrogenase A, which are closely associated with rapid tumor progression and poor prognosis. Furthermore, magnetic resonance spectroscopy (MRS) can directly detect metabolite peaks in tissues or body fluids, such as lactate, ATP, or NTP peaks, thereby offering molecular imaging evidence of tumor metabolic phenotypes and assisting in the identification of metabolically distinct tumor subtypes. More refined metabolomic profiling has revealed that lactate, polyamines, purine metabolites, and SCFAs in serum and fecal samples from CRC patients show strong correlations with molecular subtypes (e.g., CMS classification), immune cell infiltration patterns, and clinical outcomes (100).

Overall, microbiota and their metabolites not only serve as noninvasive diagnostic tools for CRC but also hold substantial promise in disease subtyping, risk stratification, and guiding personalized therapeutic decision-making.

### 7.2 Therapeutic targets and drug development

#### 7.2.1 Glycolytic targets (HK2, LDHA)

CRC cells commonly exhibit persistently high glycolytic activity, preferentially metabolizing glucose to lactate even under aerobic conditions (the Warburg effect). At the molecular level, HK2 and LDHA are key enzymes that sustain high glycolytic flux. Their upregulation not only facilitates rapid ATP production but also provides precursors for nucleotide, amino acid, and lipid biosynthesis. Certain noncoding RNAs play regulatory roles in this process; for instance, lncRNA CASC19 can bind to small nuclear ribonucleoprotein polypeptide A (SNRPA) and subsequently upregulate the transcription of HK2 and LDHA, thereby driving tumor proliferation and metabolic reprogramming.

In drug development, natural compounds (e.g., curcumin, quercetin) and several small-molecule inhibitors have been shown to directly suppress LDHA activity, shifting tumor metabolism from glycolysis toward mitochondrial OXPHOS. This metabolic switch is accompanied by increased reactive oxygen species (ROS) generation, which sensitizes tumor cells to chemotherapeutics such as 5-fluorouracil (5-FU) (18).

#### 7.2.2 Glutamine metabolism targets (GLS/CB-839)

Glutamine metabolism represents another central source of energy and carbon-nitrogen supply for CRC growth and survival.

GLS catalyzes the conversion of glutamine to glutamate, which can subsequently feed into the TCA cycle or contribute to glutathione synthesis to maintain redox homeostasis. CB-839 is an orally bioavailable, potent GLS1 inhibitor that demonstrated robust antitumor effects in PIK3CA-mutant CRC xenograft models. When combined with 5-FU, CB-839 further enhanced therapeutic efficacy by mechanisms including ROS accumulation and induction of neutrophil extracellular trap (NET) formation, ultimately disrupting tumor cell architecture (101).

Phase I/II clinical trials have shown that CB-839 in combination with the PD-1 inhibitor nivolumab is generally well tolerated; however, the objective response rate (ORR) in CRC patients was limited. Evidence suggests that CRC subtypes with high GLS dependency and concomitant NRF2 pathway activation may derive greater benefit. Furthermore, short-term GLS inhibition can enhance T-cell effector function, while prolonged inhibition may suppress immune cell activity due to substrate depletion, highlighting the need for precise patient stratification and optimization of treatment duration (102).

#### 7.2.3 Lipid metabolism and redox homeostasis

CRC cells often display a “dual-energy supply mode” with simultaneous activation of glycolysis and oxidative phosphorylation. In lipid biosynthesis pathways, high expression of key enzymes such as FASN, acetyl-CoA carboxylase (ACC), and stearoyl-CoA desaturase 1 (SCD1) supports membrane remodeling and the synthesis of signaling molecules. On the other hand, modulation of the NADPH/glutathione system plays a critical role in regulating antioxidant capacity, reducing chemoresistance, and enhancing radiosensitivity (103).

#### 7.2.4 Strategies to overcome drug resistance

There is a close link between metabolic reprogramming and therapeutic resistance. During chemotherapy (e.g., 5-FU, oxaliplatin) or targeted therapy (e.g., anti-EGFR monoclonal antibodies), CRC cells often exhibit compensatory upregulation of HK2/LDHA or GLS pathways. Combination therapies involving LDHA inhibitors or CB-839 can effectively reverse this adaptive metabolism and restore drug sensitivity. Notably, in microsatellite-stable (MSS) CRC, combining metabolic inhibitors with PD-1/PD-L1 antibodies, alongside microbiota modulation, holds promise for overcoming intrinsic immune resistance and improving responses to immunotherapy (104, 105).

### 7.3 Microbiota-based therapies and dietary interventions

In recent years, accumulating evidence has demonstrated that the development and progression of CRC are closely associated with gut microbiota dysbiosis and its metabolic pathways. The bidirectional interactions between CRC and the intestinal microecology are significant: reduction of butyrate-producing bacteria, enhanced carcinogenesis-related bile acid metabolism, enrichment of pro-inflammatory taxa, and the resulting tumorigenesis, immunosuppressive microenvironment, and therapeutic responses are highly interconnected. Consequently, interventions “targeting the microbiota” and “leveraging diet” are considered among the most plastic and translatable approaches (106, 107).

#### 7.3.1 Microbiota-related therapeutic strategies

Fecal microbiota transplantation (FMT) enables the transfer of “whole microbial communities and metabolic pathways” between donor and recipient, thereby reconstructing diversity and functional networks, enhancing immune surveillance, alleviating chemo-/radio-/immunotherapy-related toxicities, or potentially improving responses to immune checkpoint inhibitors (ICIs). However, robust evidence in CRC remains to be validated by large-scale randomized controlled trials (RCTs) (108, 109).

Multiple RCTs and meta-analyses have shown that probiotics/synbiotics significantly reduce postoperative infection rates within 30 days after CRC surgery, including surgical site infections, pulmonary, and urinary tract infections, while also improving intestinal functional recovery. Despite heterogeneity in formulations, timing, and duration, the overall direction of evidence is consistent (110). Recent studies reported that perioperative administration of *Clostridium butyricum* CBM588 promoted intestinal recovery and lowered infection risk, providing strain-specific evidence (111). During chemo-/radiotherapy, probiotics in CRC patients helped mitigate chemotherapy-related diarrhea (particularly irinotecan-induced), with potential mechanisms involving suppression of bacterial β-glucuronidase activity and reduction of intraluminal reactivation of SN-38G (112). Nevertheless, synbiotics still face limitations: strain/dose/duration remain unstandardized, and the independent benefit of synbiotics (probiotics + prebiotics) in CRC requires validation in larger cohorts. Overall, evidence consistently supports benefits in “reducing complications and improving treatment tolerance”, whereas direct evidence for “prolonged survival or tumor shrinkage” remains limited (113).

Meanwhile, microbiota-targeted strategies may also act through diet or microbial modulation to restore butyrate-producing pathways and reduce pro-inflammatory/pro-carcinogenic secondary bile acids (e.g., DCA). This represents a key approach for “correcting the metabolic microenvironment,” though current clinical evidence largely derives from combined dietary interventions with probiotics (114).

#### 7.3.2 Dietary interventions

##### 7.3.2.1 High-fiber/resistant starch (RS) and whole grains

Population-based studies demonstrate that high-fiber and whole-grain diets are associated with reduced CRC risk, partly mediated through enrichment of butyrate-producing bacteria and short-chain fatty acid (SCFA) generation, leading to lower inflammation and epithelium homeostasis. In perioperative and peritherapeutic settings, fiber-enriched enteral nutrition has shown feasibility and safety for recovery (115). The long-term CAPP2 trial in Lynch syndrome patients indicated protective effects against “non-CRC upper gastrointestinal cancers,” though CRC-specific endpoints require finer stratification by dose, duration, and baseline microbial features (116). Mechanistic and animal studies suggest RS may modulate tumor metabolism and inflammation by suppressing HK2-mediated glycolysis (117).

##### 7.3.2.2 Mediterranean diet

Epidemiological studies and systematic reviews have consistently linked adherence to MD with reduced CRC incidence (pooled HR ≈ 0.84), though evidence regarding mortality and prognosis is still accumulating. Stratified recommendations by population, tumor subtype (proximal vs distal), and treatment stage warrant further refinement (118, 119). Strong and consistent evidence indicates a dose–response relationship between red/processed meat intake and CRC risk, reinforcing the importance of replacing these with whole grains, legumes, fruits, vegetables, and unsaturated fats (120).

##### 7.3.2.3 Ketogenic diet and ketone pathways (prospective and mechanistic approaches)

Preclinical studies using murine and humanized microbiota models revealed that ketogenic diets and their key metabolite β-hydroxybutyrate (BHB) could suppress CRC growth via the HCAR2-HOPX axis, with effects linked to gut microbiota modulation. This area remains in an early mechanism-to-translation stage, and the clinical efficacy, sustainability, and safety boundaries of ketogenic interventions require validation in prospective trials (121, 122).

### 7.4 Combined strategies

1. “Diet × Microbiota (Probiotics/Synbiotics/FMT) × Cancer Therapy” combinations: Throughout surgery, chemotherapy, radiotherapy, and immunotherapy, tailored interventions may be selected according to specific goals (e.g., reducing toxicities, improving compliance/nutritional status, or potential sensitization). In the perioperative period, multi-strain probiotics/synbiotics are prioritized to reduce infectious complications; during chemotherapy (including irinotecan), suppression of β-glucuronidase and reinforcement of SCFA pathways are emphasized; during immunotherapy, high-fiber/MD diets combined with microbiota reshaping strategies (including FMT) are being explored for sensitization potential (110, 123).

2. Individualized stratification: Interventions should be matched and dynamically monitored based on baseline fecal microbiome/metabolome (e.g., *F. nucleatum* abundance, SCFA levels, bile acid profiles), tumor molecular subtypes (dMMR/MSI-H vs pMMR/MSS), host dietary habits, and metabolic status. Differential tumor microenvironment responses to microbiota modulation should be incorporated into intervention design and outcome interpretation (124).

3. Safety and standardization: FMT require rigorous donor screening and formulation standards; although bacteremia risk with probiotics in immunosuppressed patients is low, it must be monitored. Dietary interventions should avoid nutritional imbalances, with professional nutritional assessments required particularly in patients with weight loss or sarcopenia (125).

## 8 Challenges and future research directions

In recent decades, significant progress has been made in understanding the interplay between the microbiota, metabolism, and immunity in CRC. However, translating these findings into clinically applicable diagnostic and therapeutic strategies still faces multiple challenges and unresolved issues.

### 8.1 Need for deeper mechanistic insights

The contribution of microbial metabolites to metastatic “colonization” is still insufficiently defined. For instance, *Fusobacterium nucleatum* can activate TLR/NRF2 signaling and induce CYP2J2-mediated EMT, while its oxidized fatty acid products such as 12,13-EpOME may influence the metastatic niche, yet their precise role remains elusive (126). Early evidence also suggests that certain metabolites help construct “immune-privileged” microenvironments in distal organs by recruiting myeloid-derived suppressor cells and suppressing cytotoxic T-cell activity, but this hypothesis requires validation. At the same time, current studies lack single-cell resolution of the dynamic metabolic competition and cooperation between tumor and immune cells. Although emerging tools like scFEA and scMetabolism point to metabolically active macrophages in metastatic sites, a comprehensive metabolic interactome at single-cell and spatial resolution is still missing, limiting causal insights into immune suppression (127).

### 8.2 Barriers in translational medicine

Despite their potential, microbiota-targeted strategies remain difficult to standardize because patient-specific microbial compositions, metabolite profiles, and host genetics introduce large variability in treatment response. Predictive models for stratifying beneficiaries are not yet established (107). Furthermore, metabolic inhibitors such as FASN and ACC blockers demonstrate antitumor activity but also disrupt intestinal epithelial metabolism, causing toxicity and limiting tolerability. Thus, balancing efficacy with safety remains a central challenge, and current therapeutic concepts still lack robust clinical pathways for individualized interventions (103, 128).

### 8.3 Technological innovations and model requirements

Advances in spatial metabolomics and multi-omics integration are beginning to reveal metabolic–immune interactions at tissue interfaces, but applications in CRC are still limited (129).

Organoid-microbiota co-culture systems and microfluidic chip models are urgently needed to replicate the dynamic interplay among tumors, immunity, and microbial metabolism. Another unresolved issue is metabolic plasticity: tumor cells can flexibly switch between glycolysis, OXPHOS, and other pathways to escape inhibition, necessitating multi-targeted strategies (130, 131).

The development of highly selective inhibitors is also hampered by enzyme expression overlap in normal tissues, underscoring the need for tumor-specific drug delivery systems such as nanocarriers or prodrug formulations. Moreover, the absence of integrated platforms that combine metabolomics, microbiome data, and spatial omics restricts the establishment of personalized metabolic maps, while combination strategies involving microbial metabolites and immunotherapy remain exploratory and lack validation in large clinical trials (132).

### 8.4 Future directions

Future research and therapeutic strategies targeting the metabolism-microbiota-immune axis in CRC are likely to achieve breakthroughs in the following areas: Mechanistic Elucidation: Focus on delineating the roles of microbiota-derived metabolites in the “colonization” process of metastatic niches, particularly the mechanisms underlying immune suppression and the construction of metabolite niches (133). Single-Cell and Spatial Resolution: Apply single-cell RNA sequencing combined with metabolic pathway inference tools (scFEA, scMetabolism) and spatial metabolomics to precisely localize hotspots of metabolic interactions *in situ* (134, 135).

Personalized Microbial Metabolic Interventions: Predict responsive patient subgroups through metabolomics and microbiome profiling, followed by the implementation of “fecal metabolome-guided” fecal microbiota transplantation (FMT) or probiotic therapies. High-Fidelity Model Development: Establish CRC organoids or microfluidic chip models incorporating microbiota to simulate the dynamic feedback loops among tumor, immune system, and microbial metabolism (130).

Novel Drug Delivery and High-Selectivity Targets: Design gut-targeted delivery systems or inhibitors based on tumor-specific metabolic features to minimize damage to normal tissues (136, 137).

Multi-Modal Integration Platforms: Develop computational platforms capable of integrating metabolomics, spatial omics, and microbiome structural data to facilitate preclinical prediction and the design of intervention strategies.

Overall, metabolism-targeted therapy has demonstrated substantial potential in CRC, but it must overcome the challenges posed by metabolic plasticity, immune evasion, and tumor heterogeneity. Only by simultaneously advancing mechanistic studies, technological platforms, model systems, and drug development while deeply integrating the microbiota–metabolism–immune axis into precision oncology—can these strategies be successfully translated into the clinic.

## 9 Conclusion

Metabolic reprogramming in CRC represents a highly complex and plastic regulatory network, with microbiota-host co-metabolism playing a central role in reshaping energy landscapes, immune evasion, and therapeutic responses. Targeting metabolism pathways, especially when combined with immunotherapy, shows clear promise for enhancing treatment efficacy and overcoming the challenge of immunotherapy resistance, potentially benefiting patients with microsatellite-stable (MSS) CRC. The path toward precision metabolic therapy requires overcoming key challenges, including metabolic plasticity, pathway redundancy, poor selectivity of existing drugs, and adverse toxicities.

Microbiota-metabolism-immune interactions represent a crucial future entry point. Constructing individualized microbiota metabolic maps, modeling microbial co-metabolism, and integrating metabolomics with spatial immunomics will be essential for designing effective strategies that combine microbiota modulation, metabolic intervention and immune activation. Ultimately, a multidimensional therapeutic paradigm that simultaneously targets metabolism, the microbiome, and the immune system, while accounting for the spatial heterogeneity and metabolic-immune evasion mechanisms in CRC will be fundamental to advance precision medicine and improve patient outcomes.
